# Dissection of the inferior mesenteric vein versus of the inferior mesenteric artery for the genitourinary function after laparoscopic approach of rectal cancer surgery: a randomized controlled trial

**DOI:** 10.1186/s12894-019-0501-5

**Published:** 2019-08-05

**Authors:** Anna Pallisera-Lloveras, Paula Planelles-Soler, Naim Hannaoui, Laura Mora-López, Jesús Muñoz-Rodriguez, Sheila Serra-Pla, Arturo Dominguez-Garcia, Joan Prats-López, Salvador Navarro-Soto, Xavier Serra-Aracil, on behalf of Tauli-Colorectal Cancer Study Group, Anna Pallisera-Lloveras, Laura Mora-López, Sheila Serra-Pla, Salvador Navarro-Soto, Xavier Serra-Aracil, Marta Hidalgo, Paula Planelles-Soler, Naim Hannaoui, Jesús Muñoz-Rodriguez, Arturo Dominguez-Garcia, Joan Prats-López, Carmen Del Pino, Carles Pericay, Ismael Macias, Eva Ballesteros, Àlex Casalots, Eva Martinez-Bauer

**Affiliations:** 1 Coloproctology Unit, General and Digestive Surgery Department, Parc Taulí University Hospital, Sabadell, UniversitatAutònoma de Barcelona, Parc Taulí s/n. 08208 Sabadell, Barcelona, Spain; 2 Urology Department, Parc Taulí University Hospital, Sabadell, Universitat Autònoma de Barcelona, Parc Taulí s/n 08208, Sabadell, Barcelona, Spain

**Keywords:** Genitourinary dysfunction, Injury to the pelvic autonomic nerves, Total Mesorectal excision, Rectal cancer

## Abstract

**Background:**

Total Mesorectal Excision (TME) is the standard surgical technique for the treatment of rectal cancer. However, rates of sexual dysfunction ofup to 50% have been described after TME, and rates of urinary dysfunction of up to 30%. Although other factors are involved, the main cause of postoperative genitourinary dysfunction is intraoperative injury to the pelvic autonomic nerves. The risk is particularly high in the inferior mesenteric artery (IMA). The aim of this study is to compare pre- and post-TME sexual dysfunction, depending on the surgical approach usedin the inferior mesenteric vessels: either directly on the IMA, or from the inferior mesenteric vein (IMV) to the IMA.

**Methods:**

Prospective, randomized,controlled study of patients with rectal adenocarcinoma with neoadjuvant chemoradiotherapy, who will be randomly assigned to one of two groups depending on the surgical approach to the inferior mesenteric vessels. The main variable is pre- and postoperative sexual dysfunction; secondary variables are visualization and preservation of the pelvic autonomic nerves, pre- and postoperative urinary dysfunction, and pre- and postoperative quality of life. The sample will comprise 90 patients, 45 per group.

**Discussion:**

The aim is to demonstrate that the dissection route from the IMV towards the IMA favors the preservation of the pelvic autonomic nerves and thus reducesrates of sexual dysfunction post-surgery.

**Trial registration:**

Ethical and Clinical Research Committee, Parc Taulí University Hospital: ID 017/315. ClinicalTrials.gov TAU-RECTALNERV-PRESERV-2018 (TRN: NCT03520088) (Date of registration 04/03/2018).

## Background

Despite the advances in adjuvant and neoadjuvant chemotherapy (QT) and radiotherapy (RT) for colorectal cancer, surgery remains the only curative treatment [1]. Since its first description by Heald in 1982 [2, 3], Total Mesorectal Excision (TME) has been the standard surgical technique for the treatment of rectal cancer, and has brought about significant improvement in oncological results in terms of both survival and local recurrence [4].

However, the literature describes post-TME rates of urinary dysfunction (urinary incontinence, difficulty in voiding or neurogenic bladder) of up to 30% [5, 6]. The prevalence of sexual dysfunction is above 50%; in men, it includeserectile dysfunction and ejaculation problems, and in women, decreased vaginal lubrication, dyspareunia and difficulty reaching orgasm [5, 6]. For this reason, the study of functional results such as genitourinary function, fecal continence and quality of life in general is taking on increasing importance [1].

Although other factors may be responsible for postoperative genitourinary dysfunction, the main cause is intraoperative injury to the pelvic autonomic nerves [1, 6]. This inadvertent lesion usually occurs due to a lack of anatomical knowledge or due to poor visualization of the nerves [6]. Minimally invasive techniques such as laparoscopic colorectal surgery and robotic surgery have improved surgical technique and surgical visibility, that helps in recognizing the inferior hypogastric plexus [7]. Moreover, a thorough acquaintance with anatomical reference points and experience ofthe dissection during TME reduces the risk of injuring the autonomic nerves, and leaves the mesorectal fascia intact. Indeed, proficient TME technique helps to preserve the autonomic nerves, lowering the incidence of urinary dysfunction from 10 to 30% to 0–12% and sexual dysfunction from 40 to 60% to 10–35% [5, 6]. Table 1 shows the genitourinary dysfunction after TME surgery, with the preservation of the pelvic autonomic nerves [8–10].

**Table 1:** Genitourinary dysfunction after TME surgery with preservation of the pelvic autonomic nerves

Author (year)	Surgical approach	N	Measurement methods	Time of measurement	Urinary dysfunction (%)	Sexual dysfunction (%)
Dulskas A, et al. [1] (2016)	Open	108	Urinary: IPSS, BFLUTS Sexual: IIEF, FSFI	Pre-IQ and 6 m postIQ	Men 8.3% Women 11.1%	Men 27.8% Women 11.1%
Pontallier A, et al. [7] (2016)	Lap	34	Urinary: IPSS Sexual: IIEF, FSFI	12 months after closure of the stoma	23.5%	61%
	Transanal	38			31.5%	29%
Liu Z, et al. [8] (2016)	Lap	518	Own scale	At discharge and 6 months postIQ	NE	Ejaculation 42.5% Erection 41.9%
Adam JP, et al. [9] (2016)	Lap	169	Urinary: IPSS Sexual: IIEF, FSFI	Pre-treatment, post-QTRT and 3,6 and 12 months postIQ	17%	Men: Erection 43% Ejaculation 68% Women: Lubrication 62% Dyspareunia 42% Orgasm 57%

*TME* total mesorectal excision, *SP* surgical procedure, *IPSS* International Prostatic Symptom Score, *BFLUTS* Bristol Female Lower Urinary Tract Symptoms, *IIEF* International Index of Erectile Function, *FSFI* Female Sexual Function Index. QTRT: chemoradiotherapy

**Fig. 1:**
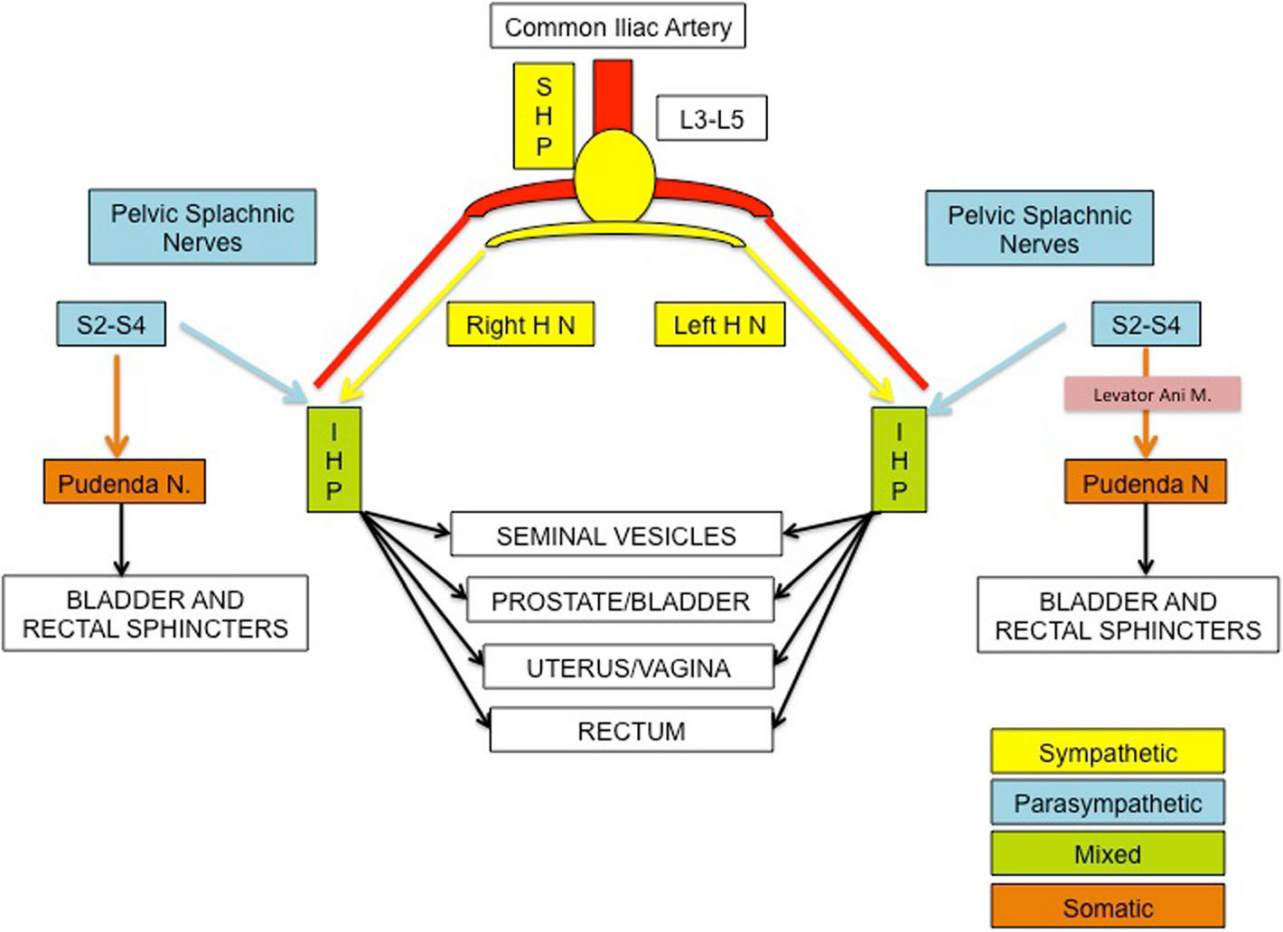
Outline of the pelvic autonomic nerves and their target organs

In rectal cancer surgery particular attention must be paid to certain areas, due to the risk of autonomic nerve damage [6]. Figure 1 depicts the surgical anatomy of the pelvic region and the key points where the risk of injury increases during surgery (Table 2) [5, 6, 11–14]. As described in the table, one of the regions with the highest risk of injury is the inferior mesenteric artery (IMA).

**Table 2:** Areas with risk of lesion in the main autonomic nerves, and their effects

Nerve injured		Area of risk	Urinary dysf.	Sexual dysf.
SHP (sympathetic)		Dissection and ligature of IMA	Stress urinary incontinence	Retrograde ejaculation
Hypogastric nerves		Posterior dissection		Alteration in ejaculation (no alteration erection)
Splanchnic nerves (parasympathetic)		Rare in TME In radical lymph node dissections	Neurogenic bladder	Men: alteration erection Women: Dyspareunia
IHP (mixed)		Lateral dissection	Incontinence Difficulty voiding	Problems with ejaculation and erection
- IHP one-sided			Difficulty voiding	Men: Ejaculation and erection problems Women: Problems with genital lubrication
- IHP two-sided			Bladder denervation	Men: impotence Women: dyspareunia and ↓ability to reach orgasm
Neurovascular bundle of Walsh and cavernous nerve		Anterior dissection		Men: impotencia and erectile dysfunction Women:dispareunia and reduced genital lubrication
Pudendo nerve and levator ani		Perineal stage of Miles APR	Urinary incontinence	Sexual sensorial impotence

Recently, Melani [15] described a new laparoscopic approach for the release of the splenic flexure, in which a dissection of the mesenteric vessels is performed from the inferior mesenteric vein (IMV) to the IMA. This route presents a dissection plane that follows the anatomical peritoneal sheets of the retroperitoneum and improves the visualization of the pelvic autonomic nerves from their origin. Compared with the standard approach to the inferior mesenteric vessels (i.e., directly on the IMA), we believe thatthe approach from the IMV to the IMA will improve nerve preservation and thus reduce the incidence of sexual and urinary dysfunction.

## Hypothesis and objectives

### Hypothesis

The hypothesis is that the dissection route from the IMV to the IMA favors the preservation of the pelvic autonomic nerves, and thus reduces genitourinary dysfunction in patients after laparoscopic TME.

### Objectives

The main objective is to determine the pre- and post-TME rates of sexual dysfunction in patients with rectal cancer randomly assigned to one of two nerve preservation techniques.

In both groups, the secondary objectives are to assess: urinary dysfunction pre- and post-TME; the visualization and preservation of the superior hypogastric plexus (SHP), the hypogastric nerves, the inferior hypogastric plexus (IHP) and the Bundle of Walsh; and the impact of genitourinary dysfunction on patients’ quality of life.

## Methods/design

### Study design

In this prospective, controlled randomized study of patients undergoing laparoscopic TME for rectal cancer, with neoadjuvant chemoradiotherapy, patients will be assigned to one of two groups, in a 1:1 ratio: the intervention arm (dissection route from the IMV to the IMA), and the control arm (dissection route directly to the IMA).

### Study area and participants

The study will be carried out at the ParcTaulí University Hospital of Sabadell (Sabadell, Barcelona, Spain), which has a specialized unit for Colorectal Surgery, Urology, Radiodiagnosis, Oncology, and Radiotherapy.

The study population will comprise patients diagnosed with rectal cancerundergoing total colonoscopy with multifocal biopsy, rectoscopy with endorectal ultrasound (ERUS), pelvic magnetic resonance imaging (MRI) and thoraco-abdominal computed tomography (CT). All patients will be evaluated by the multi-disciplinary committee of colorectal tumors and must comply with the following criteria:

### Inclusion criteria

Male sex; age 18 years or over; diagnosis of rectal adenocarcinoma ≤10 cm from the anal verge, measured by rigid rectoscopy, candidate to TME; candidate for neoadjuvant treatment (chemoradiotherapy); scheduled laparoscopic radical TME surgery carried out by colorectal surgeons; ASA I, II or III; provision of signed informed consent.

### Exclusion criteria

Female sex; age under 18; not candidate for neoadjuvant treatment and/or adenocarcinoma > 10 cm from the anal verge candidate to partial TME; abdominoperineal resection (APR); emergency surgery; recurrent tumor; cT4; history of infra-abdominal or pelvic prostate procedures; radiotherapy prior to the current process; patients with severe sexual or urinary dysfunction before surgery; no provision of signed informed consent.

### Criteria for withdrawal

Patients who do not attend the subsequent follow-up.

### Definitions

Sexual dysfunction (SD) is defined as difficulty at any stage of sexual intercourse (which includes desire, arousal, orgasm and resolution) that prevents the individual or their partner from enjoyingsexual activity [16]. Erectile dysfunction (ED) is defined as the persistent inability to reach and maintain an erection sufficient to allow satisfactory sexual performance, which mayhave a physical and psychological effect on health and have a significant impact on the quality of life of patients and their respective partners [17].

According to the *2017 European* Association of *Urology* (EAU) *guidelines*, the diagnostic evaluation should be carried out with a correct anamnesis, the use ofvalidated questionnaires such as the International Index of Erectile Function or IIEF-5[18] and the Erection Hardness Score (EHS) [19], a full physical examination and basic analytical parameters such as total testosterone levels [17]. In addition, Doppler ultrasound of the penis is recommended, with the aim of recording objective parameters of induced erection so as to identify the cause of ED [17].

The IIEF-5 questionnaire consists of five questions that are scored from 0 to 5. Scores of 21 or lower are considered to indicateED. The test also classifies the severity of the ED as mild (score 17–21), mild to moderate (score 12–16), moderate 8–11 or severe (score 1–7) [18].

The EHS classifies erections in four grades: grade 1 or severe (does not allow penetration); grade 2 or moderate (increased circumference, relatively rigid, but not enough to achieve penetration); grade 3 or mild (increased length and circumference to allow penetration, but not enough to be satisfactory); grade 4 (maximum dimensions in terms of length and strength to achieve satisfactory penetration) [19].

Urinary or voiding dysfunction is defined as any alteration in the correct functioning in the storage or periodic emptying of urine, either in isolation or simultaneously [20]. To measure the severity of symptoms and the impact on the quality of life, or to monitor the response to a particular treatment, the ICIQ-SF (International Consultation on Incontinence Questionnaire) is used [21]. The ICIQ-SF consists of three questions, with a total score of 1 to 21; a score higher than 0 is considered a diagnosis of urinary incontinence.

The IPSS (International Prostate Syndrome Score) [22] consists of seven questions that assess voiding, frequency, intermittency, urgency, weak stream and effort. Each question is scored from 0 to 5: an overall score of 1 to 7 reflects mild symptomatology, one from 8 to 19 moderate, and from 20 to 35 severe. However, the only method that can objectively evaluate the filling and emptying phase, and therefore diagnose the presence and type of urinary dysfunction, is the urodynamic study [20].

### Informed consent and legal considerations

Patients will be informed, both orally and in writing, of the objective and duration of the study. It will be made clear that participation is voluntary and they will be informed of all the possible risks and associated complications. Patients who meet the inclusion criteria and sign informed consent will enter the randomization process. They may withdraw their consent to participate at any time.

The variables described are recorded in a computerized database (Microsoft® Acces 2003). The data will be introduced in a protected format to avoid (as far as possible) the entry of out-of-range or anomalous values. The patients’ data will berecorded anonymously and in accordance with the Spanish legislation on data protection (LOPD, 1999) [23].

The study protocol, the patients’data and the informed consent documents have been approved by the Clinical Research Ethics Committee in accordance with the 2015 Royal Decree on medical trials with drugs. The Parc Taulí’s ethics committee will serve as the reference (ID: 2017/315). The study has been registered in the ClinicalTrials.gov database (ID: NCT03520088).

The trial will be carried out in accordance with the 7th Revision of the Declaration of Helsinki [24] and follows the guidelines of the 2013 SPIRIT Standard Protocols for Clinical Trials [25],CONSORT 2010 for Clinical Trials [26],and the Spanish Agency for Medicines and Health Medical Products).

### Description of the randomization

A simple randomization system will be used, in which the selected patients will be randomly assigned by a computer program in a 1:1 ratio to one of two study arms: the intervention arm (dissection route from the IMV to the IMA) and the control arm (dissection route directly to the IMA).

### Blinding

Neither the patient nor the urologist performing the sexual and urinary function study will have access to any surgical data; nor will they know which arm of the study the patient belongs to.

### Trial interventions

**Fig. 2:**
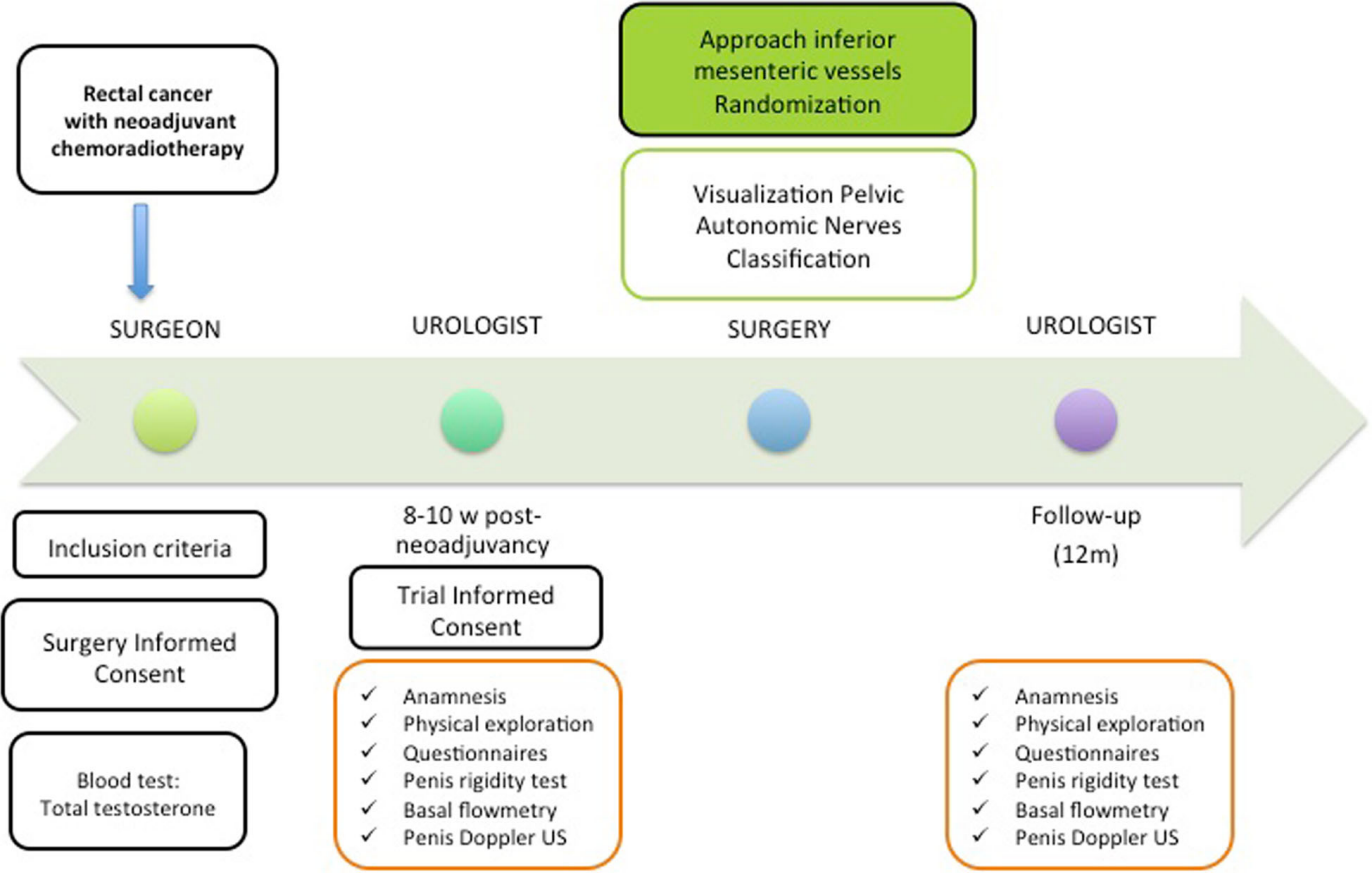
Proposed study schedule

Patients who meet the inclusion criteria and sign the informed consent document will be referred to the urologist for functional study after 8–10 weeks of neoadjuvant treatment, before surgery (Fig. 2).

The study consists of: a) an anamnesis with medical and psychosexual history; b) total testosterone level (ng/dL); c) Erection Hardness Score (EHS) [19]; d)IIEF-5 [18];e) I-PSS [22]; f)ICIQ-SF [21]; g) the European Organization for Research and Treatment of Cancer Quality-of-Life questionnaire (EORTC-QLQ C30) [27]; h) Doppler ultrasound of the penis; i) Urodynamic study.

For the Doppler ultrasound of the penis, a baseline study will be carried out to assess the ultrasound structure of the scrotal wall, both corpora cavernosa and the corpus spongiosum, the presence of calcifications or plaques of fibrosis, and their location. Subsequently, 15 mg of prostaglandin E1, a vasodilator agent that induces erection,will be applied to one of the corpora cavernosa (the sidechosen for the injection is irrelevant). After 20 min of the application the ultrasound provides dorsal and transversal measurements during the initial phases, and oblique-longitudinal measurements once tumescence begins, of the diameter of both cavernous arteries, their peak systolic flow velocity (cm/s) and their flow velocity at the end of diastole (cm/s) [28].

For the urodynamic study a flowmetry will be performed, recording: maximum urine volume (mL), maximum flow (mL/s), average flow (mL/s), and urination time (s)) and subsequently an ultrasound evaluation of postvoid residue (mL) [20].

Subsequently, patients undergo surgery after being randomly assigned to one of the two study arms. Before surgery, all patients will undergo mechanical colon preparation with antibiotic and thromboembolic prophylaxis, in accordance with the center’s protocol.

### Surgical technique

The surgery performed will be TME, using laparoscopic low anterior resection (LAR) approach or transanal TME (TaTME) [29], combining a laparoscopic approach and Transanal endoscopic operation (TEO).

The surgery is performed only by 5 surgeons, who are part of the colorectal unit. All of them experts in laparoscopic surgery and they have training on both techniques (intervention and control arm).

The surgery is performed laparoscopically with the patient in the Lloyd-Davies position. If release and mobilization of the splenic angle is necessary the Melani technique is applied [15], with the subsequent dissection of the IMA according to the randomization schedule, as follows:

- Control group, with direct dissection of the IMA. Opening of the mesosigma peritoneum, laterally separating the SHP and hypogastric nerves. Visualization and preservation of the ureter, left primitive iliac vessels and the left gonadal vessels, and maintaining Gerota’s fascia intact. High ligation of the IMA is performed about 1.5-2 cm from its origin in the aorta. The medial-to-lateral dissection proceeds, as far as the dissection of the splenic angle mobilization;
- Intervention group, with dissection of the IMV to the IMA. Release of the splenic angle is followed by medial to lateral dissection from the IMV to the IMA, and a high ligation is performed about 1.5-2 cm from its origin in the aorta. Subsequently, the dissection follows the plane of the retroperitoneum from cranial to caudal and medial to lateral as far as the mesosigma.

The surgery continues with the following standardized steps: opening of the left Told’s Fascia; mesorectal dissection immediately posterior to the superior rectal artery, following the plane anterior to the presacral parietal fascia; bilateral dissection, avoiding traction and blunt dissection that might damage the nerves or the integrity of the mesorectum; the anterior dissection is performed after completing the posterior dissection as far as the pelvic floor, leaving the Denonvilliers fascia intact, and following the avascular plane.

In the case of laparoscopic LAR, a Pfannenstiel incision is made toextract the specimen with the subsequent mechanical end-to-end colorectal anastomosis with EEA™ Auto suture 28 (Covidien™, Dublin, Ireland).

In transanal TME [29],TEO equipment is used (Karl Storz GmbH, Tüttlingen, Germany), and a pouch is made to close the distal rectum at least 1 cm below the visualization of the lesion. The area of the sectionis marked 1 cm from the pouch and transanal dissection of the mesorectal is carried out until reaching the dissection performed by laparoscopy. The lateral anterior dissection continues, leaving the Denonvilliers fascia intact.

In the case of an end colostomy is required, a colostomy orifice is performed in a previously marked site, sectioning the proximal colon with a scalpel and performing an end colostomy, with placement of a prophylactic mesh using the modified Sugarbaker technique [30, 31].

### Follow-up

The patient will be referred again to the urologist to repeat the genitourinary dysfunction study 12 months after surgery: Doppler ultrasound of the penis, baseline flowmetry, EHS and the IIEF-5, I- PSS, ICIQ-SF and EORTC-QLQ C30 questionnaires.

### Study variables

#### Main variable

Preoperative, post-neoadjuvant and postoperative sexual dysfunction at 12 months in all intervened patients will be determined by the urologist, based on the results of the IIEF-5 questionnaire and/or Doppler ultrasound (peak systolic velocity, tele-diastolic velocity and response to vasodilator agent). Scores of 21 or below on the IIEF-5 questionnaire will be taken to indicate sexual dysfunction. The ultrasound will confirm or rule out the presence of sexual dysfunction generated by the test: an adequate response to thevasodilator agent, with blood flow above 35 cm/s in a patient with an altered IIEF-5 or EHS test will be considered a diagnostic criterion for ED of a neurogenic cause.

#### Secondary variables

- Preoperative, post-neoadjuvant and postoperative erection hardness at 12 months;
- Preoperative, post-neoadjuvant and postoperative urinary dysfunction at 12 months, determined based on the results of the ICIQ-SF, IPSS and baseline flowmetry questionnaires (maximum urine volume, maximum flow, mean flow and urination time) with postvoid residue (mL). The presence of urinary dysfunction will be suspected if the patient has a score of 1 or aboveon the ICIQ-SF questionnaire, a score of 7 or above in the IPSS questionnaire or alterations in the flowmetry parameters: a maximum flow below 15 mL/s, a urinary volume less than 150 mL and/or a postvoid residue greater than 20% will be considered abnormal.
- Quality of life post-neoadjuvancy and 12 months after surgery will be assessed using the EORTC QLQ-C30 questionnaire.
- Visualization and preservation of the SHP, hypogastric nerves, the IHP and the neurovascular bundle of Walsh, during surgery (for each of these nerves), classified into: a) visualization and preservation of the nerves; b) visualization but no preservation; c) no visualization. This variable will be determined by the surgical team that performs the procedure. It will be taken into account that some patients may need the nerve resection for oncological purposes. The intervention will be recorded so that a surgeon not participating in the procedure can classify the nerve injury, as described above, and independently from the main surgeon. If there is disagreement between the two surgeons, the evaluation of a third external reviewer will be requested.

### Other variables

Preoperative: age (years), body mass index (BMI) (kg/m2), smoking (packets/year), Charlson comorbidity index (CCI), previous pelvic surgeries, previous radiotherapy, Briganti criteria (low /intermediate/high risk), total testosterone levels (ng/dL). Characteristics of the tumor: height of the lesion (cm) by rectoscopy, location of the tumor (anterior, posterior, right or left lateral), cTNM staging.

#### Surgical

Type of surgical technique (LAR, NOTES-hybrid), surgical time.

#### Postoperative

Morbidity at 30 days (according to the Clavien-Dindo classification [32]), 30-day mortality, CCI [33], hospital stay. Definitive pathological anatomy: Size of the lesion (mm), pTNM and the completeness of mesorectal excision,according to the Quirke classification.

### Statistical analysis

#### Sample size

With an α risk of 0.05 (5%), a one-sided hypothesis and a βriskof 0.1 (power of 90%), taking into account that the prevalence of post-TME sexual dysfunction is 50% and intending to reduce the figure to 20%,it will be necessary to include 41 patients in the two groups. Estimating a possible loss to follow-up of 10%, the total sample required will be 90 patients: 45 per group.

In our hospital rectal surgery for cancer is expected between 60 and 70/year, with inclusion criteria, we think that about 45–50 could be included. That means, that probabibly in two years, the recruitment should be finished.

#### Analysis of patients by intention to treat, per protocol, and patients lost

Patients will be analysed by intention to treat (ITT), per protocol, andlost patients.

The intention to treat analysis will be applied to all randomized patients.

The per protocol analysis will include part of the group in which direct dissection of the IMA is performed, and all the group that undergo dissection from the IMV to the IMA. Statistical analysis of the lost patients will be performed.

#### Monitoring

Monitoring will be carried out by a data manager. The variables will be recorded on an Access database which the data manager will manage via the various hospitals.

#### Statistical analysis

To evaluate the main objective of the study, the reduction in sexual dysfunction achieved by the preservation of the pelvic autonomic nerves, we will use the method based on the one-sided confidence interval at 95% of the difference in sexual dysfunction between the two groups [34, 35], in both the intention to treat and per protocol samples.

The quantitative variables will be described giving values of mean and standard deviation, or medians, interquartile interval and range, as appropriate. The categorical variables will be described in absolute numbers and percentages.

The statistical analysis of the quantitative variables, with independent groups, will be performed with the parametric Student’s T-test, provided that its conditions of application are met; otherwise, the non-parametric Mann-Whitney U test will be used.

In the statistical analysis for categorical variables, the Pearson Χ^2^ test or the Fisher exact test will be used.

These methods will be used to compare the baseline characteristicsof the two groups in order to assess whether the randomization has been effective.

Statistical significance will be set at a *p* value of less than 0.05.

## Discussion

In our study, only men, submitted to TME and candidate for neoadjuvant treatment (chemoradiotherapy) are included, so that the sample is more homogeneous in terms of final results.

TME, performed by minimally invasive surgery through laparoscopic or robotic approaches and/or TaTME, reduces morbidity and accelerates postoperative recovery. Improved quality of life in the medium and long term continues to be one of the main objectives of TME.

Some authors have shown better functional results with the robotic approach due to the superior movements of the wristed instruments that facilitate fine dissection, coupled with a stable and magnified view that helps in recognizing the inferior hypogastric plexus [7]. Due to the lack of experience in robotic surgery and our long experience in laparoscopic surgery, the design of our study has been made only in laparoscopic approach, to avoid the learning curve bias.

It is known, that quality of life and sexual dysfunction rates are not similar among patients submitted to LAR and APR. In our study, only patients submitted to LAR are included, in order to have a more homogeneous sample.

Different definitions of the concept of “quality of life” have been proposed and perhaps no single definition is fully satisfactory. The best one may be: “a state of general satisfaction, derived from the realization of the potential of the person. It has subjective aspects and objective aspects. It is a subjective feeling of physical, psychological and social well-being. It includes, as subjective aspects, intimacy, emotional expression, perceived safety, personal productivity and objective health. As objective aspects, material well-being, harmonious relationships with the physical and social environment and with the community, and health objectively perceived”. As early as 1986, the European Organization for Research and Treatment of Cancer (EORTC) launched a program to assess the quality of life of patients participating in international clinical trials. This is why we have chosen the questionnaire validated by this organization,the EORTC-QLQ C30 [27], to evaluate the quality of life in our study.

Quality of life in our patients is defined by the need for an ostomy orby the presence ofsexual and urinary dysfunctions. Data on these dysfunctions are generally compiled through the use of questionnaires, which all present subjective limitations. As described above, sexual dysfunction is the difficulty at any stage of sexual intercourse (including desire, arousal, orgasm and resolution) that prevents the individual or their partner from enjoying sexual activity [5]. As detailed in the material and methods section, we have followed the indications of the EAU *guidelines (2017)* for the study of sexual dysfunction. The reason for adding the Doppler ultrasound study is to evaluate the cavernous arteries and their spectral evolution, in order to record objective parameters of induced erection and thus to identify the cause of the erectile dysfunction [17]. In addition, there is no reference in the literature of the study of SD in TME with the Doppler and that will give us objective data about it. Clearly, anxiety, psychological strain and any adrenergic stimulus may lead to false positive results, but with the exception of these cases, an altered result can be attributed to a neurogenic cause of erectile dysfunction in patients with a history of nerve damage [36]. Urinary dysfunction is defined as any alteration in the correct functioning in the storage or periodic voiding of urine, either in isolation or simultaneously. The tests used in the study are, once again, the ones recommended in the EAU *guidelines*; however, the only method able to objectively evaluate the presence and type of urinary dysfunction is urodynamic study by means of flowmetry [20].

The rationale for our study is the belief that the dissection routefrom the IMV to the IMA can improve nerve preservation of the nerves. The reason is anatomical, because this approach route follows the plane of dissection of the peritoneal sheets of the retroperitoneum, favoring visualization (and therefore a better preservation) of the pelvic autonomic nerves from their origin.

We hope that our study will show that the approach from the IMV to the IMA can increase the preservation of the pelvic autonomic nerves, and can thuslower the rates of sexual dysfunctionto a figure around 20%.

In parallel, though this is not the objective of our study, we believe that the detection of possible pre-surgical sexual dysfunction can promote prompt treatment of this dysfunction after surgery. Previous studies in the setting of prostate adenocarcinoma surgery have shown that the selection of the patient (according to age and preoperative sexual function, measured with the IIEF), as well as the surgical technique (in the case of radical prostatectomies, the preservation of the neurovascular bundles) are the main determinants of postoperative erectile function [37]. Correct stratification of patients is vital to patient assessment and to the selection of the most appropriate postoperative treatment strategy. These studies conclude that when patients are correctly stratified and selected, a high recovery rate of erectile function can be expected after radical prostatectomy with neurovascular preservation.

If our hypothesis is supported, we believe that the change in approach proposed here will help to improve quality of life in patients undergoing surgery for rectal cancer with neoadjuvant radiotherapy.

## Data Availability

Not applicable.
